# Assessing the Implementation of a Behavior Change Intervention: Process Evaluation of a Stepped-Wedge Cluster Randomized Controlled Trial for Newborn Health

**DOI:** 10.3390/healthcare8020187

**Published:** 2020-06-26

**Authors:** Alessandra N. Bazzano, Jeni A. Stolow, Ryan Duggal, Richard A. Oberhelman, Yaoyao Sun, Chivorn Var

**Affiliations:** 1 Department of Global Community Health and Behavioral Sciences, Tulane School Public Health and Tropical Medicine, New Orleans, 70112 LA, USA; jstolow@tulane.edu (J.A.S.); oberhel@tulane.edu (R.A.O.); ysun21@tulane.edu (Y.S.); 2 School of Medicine, Tulane University, New Orleans, 70112 LA, USA; rduggal@tulane.edu; 3 School of Nursing, Shandong University, Shandong 250012, China; 4 Reproductive Health Association of Cambodia, Phnom Penh 905, Cambodia; chivorn@rhac.org.kh; 5 National Institute of Public Health, #2 Kim Y Sung Blvd, Tuol Kork, Phnom Penh 1300, Cambodia

**Keywords:** newborn, implementation research, process evaluation, qualitative, behavior change

## Abstract

Maternal and under five-year-old mortality rates have reduced in the last two decades globally due to concerted effort, yet newborn deaths remain unacceptably prevalent. Behavior change is an important component of interventions to address newborn health problems in low-income countries. In Cambodia, maternal and newborn mortality has markedly decreased, and continued improvements will allow the country to achieve further reduction in newborn morbidity. The results of an implementation study of the Newborn Care and Infection Control Initiative using process evaluation are presented to provide insight into the trial implementation and context of the program that may have contributed to intervention results. The study utilized a mixed method process to explore the context, mechanisms, and implementation of intervention components: training of village health support group volunteers to provide home visits, training of midwives on infection prevention and control around the perinatal period, counseling on newborn care, and provision of training materials for counseling and intervention. Implementation was evaluated through quantitative and qualitative data collection including surveys, observation, semi-structured interviews, focus groups, and visual media. Descriptive statistics summarized the quantitative data and thematic analysis was used to explore the qualitative data. The evaluation identified several factors that might have influenced the outcomes of the trial: continuity of health center staff communication, timing and ability to complete home visits, and training quality. Additional support for parents in the perinatal period, preferably provided at the community level, will contribute to further improvement in health outcomes for newborns in this area. Researchers in this context should consider mechanisms to improve the coordination of health facility staff counseling while providing support and resources to ensure home visits to families with newborns are made on time. Attention to staffing, training, and quality of newborn health interventions is critical in planning for the scaling-up of newborn health programming.

## 1. Introduction

Maternal and child health outcomes have been improving in Cambodia over the past several decades. Although significant improvement has been made in maternal and under five-year-old survival, advances in reducing newborn mortality have been slower. Nonetheless, the overall country rate of neonatal mortality has gone from 40/1000 live births in 1996 to 16/1000 live births on average in 2016 [1]. While overall rates have been decreasing, progress has been uneven. The most recent DHS data indicated that significant regional disparities exist such that neonatal mortality is much higher in certain provinces, which are generally more remote and where health services lag behind, ranging from 12 per 1000 live births in Battambang/Pailin province to 36 per 1000 live births in Mondul Kiri/Ratanak Kiri province [2]. As noted in a recent UNICEF report, there continue to be areas in Cambodia where vulnerable groups are left behind, for example, rural versus urban families, and among families with higher as compared with lower maternal education [3].

Interventions targeting quality of care and infection prevention and control are considered key for improving health outcomes during delivery and in the early postnatal period [4]. Evidence in the literature highlights the importance of infection prevention, handwashing, and perinatal water sanitation hygiene (WASH) as important areas for intervention. One systematic review focusing on evidence for clean birth and postnatal care practices identified maternal handwashing and cord care as associated with a reduction in all cause neonatal mortality [5]. A neonatal intervention study in Bangladesh described community-based chlorhexidine for umbilical cord cleaning compared with other umbilical cord-care regimens [6].

Several indicators have been targeted for improvement in Cambodia, including increasing the proportion of women attending at least four antenatal care visits, visiting healthcare providers early in pregnancy, delivering with a skilled birth attendant, and delivering at health facilities [7]. Qualitative work in Cambodia has also identified the need for skilled birth attendants during labor, birth, and the immediate postpartum period as a means of improving neonatal outcomes [8].

The Newborn Infection Control and Care Initiative (NICCI), a stepped wedge cluster randomized trial, was implemented in Takeo, Cambodia from February 2015 to November 2016 to test the effect of a linked community–facility intervention to improve newborn health in the community, and detailed information on the trial methodology and outcomes are published elsewhere [9,10]. In brief, the intervention consisted of a two-pronged approach targeting: (1) quality of care at the health facility level where women deliver—Health Center (HC)—through refresher training on infection prevention control and postnatal counseling, and (2) at the community level, improving awareness of newborn health care and care-seeking through postnatal home visits carried out by volunteer health workers. The aims were to improve care provided in facilities where women deliver and to improve recognition of special care and attention needed for newborns at home in the first week following birth, as the majority of newborn deaths occur early in the newborn period. Community health workers in Cambodia consist of village health support group volunteers (VHSGs), functioning as a de facto outreach program for various health initiatives. The volunteers do not receive ongoing training but rather are trained and remunerated according to specific initiatives to mobilize the cadres. Access to health care is one of the most prominent health issues associated with poverty in Cambodia [11]. VHSGs were established as a tool to bridge the gaps in access between these communities and the health centers by improving communication and collaboration [12].

The stepped wedge trial consisted of the intervention being introduced in a new HC and catchment area (considered a cluster) each month until all catchment areas transitioned from control to intervention. The trial was designed to incorporate a predefined process evaluation in order to better understand factors related to trial implementation and community acceptance. Process evaluations related to improving maternal and newborn health have been conducted for similar studies in order to assess implementation [4,13,14,15], and are considered an important means of understanding the relative success of large, community-based randomized trials.

Conducting a process evaluation creates an opportunity for researchers to gain insight into the context, mechanism, participant perceptions, and the implementation process of an intervention. A process evaluation can aid in explaining the outcomes of an intervention [14,16]. A key benefit of a process evaluation is that it allows implementers to determine if an apparent failure of an intervention was due to the intervention concept or the intervention implementation [17]. Likewise, a process evaluation allows insight as to which aspects of the intervention were more or less effective and what should be carried on to future programming at scale. The importance of process evaluations specifically tailored for randomized control trials (RCTs) has also been documented [13]. Process evaluations for RCTs have been utilized to differentiate strengths and weaknesses among the components of the intervention, investigate contextual factors that affect an intervention or its outcomes, assess the reach of the intervention, and study the way outcomes vary among subgroups or locations [18]. Process evaluations are also considered useful when assessing a complex randomized intervention [19]. A process evaluation can aid in assessing possible inconsistencies with implementation across sites, or inconsistences in resources and personnel across sites.

The current study sought to explore the implementation, context, mechanisms, and acceptability of the NICCI intervention through a mixed methods process evaluation study utilizing mixed methods data collection and analysis.

## 2. Methods

Quantitative monitoring data were collected throughout the trial implementation at six monthly intervals over three rounds. Data collection included abstraction from records of attendance sheets collected at trainings, checklists completed every two months by VHSG coordinators using spot-checks, completion of training material checklists at each health facility, and six-month follow-up forms.

Qualitative data were collected during the second half of the trial. Data collection included in-depth interviews with mothers, VHSG volunteers, and VHSG coordinators (who were employed by the study team), as well as focus group discussions with health center staff. Purposive sampling was utilized to select interview participants who had lived experience with the phenomena of interest, in this case the intervention. Sample size was chosen based on logistical resources and feasibility of data collection during an ongoing intervention study. The qualitative data collection consisted of thirteen interviews with VHSGs, fifteen interviews with mothers, and one focus group with fifteen VHSGs. Interviews with VHSGs and mothers took place in and around their homes (allowing for privacy), and the focus group took place at the health center. Interview guides were pretested and then used to guide qualitative data collection. All interviewers were trained in data collection and translation was provided by native speakers of the local language. Interviews were audio recorded and field notes taken at time of interview. Afterward, notes taken by the interviewers were incorporated into full transcripts with a review of the audio recordings.

Qualitative data were synthesized via inductive content analysis [20]. The approach to analysis and synthesis was guided by an open and reflective lifeworld research paradigm emerging from phenomenology [21]. Analysis was undertaken by two experienced qualitative researchers, and included independent open coding, followed by discussions to reach consensus on coding and themes. The NVivo software was utilized for the qualitative analysis. Data review and examination were structured by the components of the study for process evaluation, and evolved as themes emerged from the data. Analysis steps included: (1) familiarization with transcripts; (2) iterative development of a coding framework which identified key components of the program to be evaluated; (3) coding all transcripts via the NVivo software; (4) matching the findings of the coding process context; and (5) interpreting findings of the thematic analysis for meaning and synthesis. Once the analysis was complete, the team reflected on how the actual implementation and outcomes matched the model initially created by the research team.

Quantitative analysis consisted of calculating descriptive statistics and frequencies, and by compiling information collected on a routine basis by the intervention coordination team, in order to assess implementation.

The ethics approval for this study was granted through Tulane University IRB approval number 534777-4 and also through the Cambodian National Ethics Committee for Health Research IRB number NECHR397.

## 3. Results

The NICCI trial and process evaluation took place from February 2015 to November 2016. Table 1, Table 2 and Table 3 provide demographic data on participants included in the process evaluation and qualitative data collection. In Table 1, characteristics of VHSG health volunteer interview participants are described. All VHSG interview participants had employment outside of their volunteer position and four had multiple types of employment. The majority of the VHSGs were female (10 of 13) and the mean age of volunteers was 47, with only five years of education. Experience of volunteers ranged widely, with one having only been working for one year, while another had been serving as a volunteer for over two decades.

Table 2 presents the characteristics of the focus group discussion (FGD) participants, all of whom were midwives from local health centers, trained at either the primary or secondary level. Primary midwifery training includes one year of training following completion of at least seven years of education. Secondary midwifery training is more advanced and consists of four years of training. Of those who participated in the FGD, the mean age of midwives was 32, mean years of education was 11, and all were female.

In-depth interviews were conducted with mothers from the study area who had recently given birth. All but three mothers reported some form of occupation outside the home, and some mothers reported multiple occupations. The majority of mothers (*n* = 12) had a minimum of five years of education and were under 30 years of age (*n* = 13). The characteristic “ID Poor Card” refers to a special designation by the administrative government for health insurance and other social support coverage for families defined as meeting criteria related to poverty (as measured by assets). In addition to general demographic data, information was collected on mothers about whether their families had been asked to clean the delivery room themselves following the mothers most recent and (if applicable) other deliveries prior to the most recent birth. This information related to infection prevention control training delivered to midwives during the study, where it was recommended that families of laboring women not be responsible for cleaning delivery rooms in lieu of janitorial staff or other health facility staff.

Table 4 presents a summary of the quantitative data analyzed for the process evaluation. Results of the quantitative data analysis of the trial implementation outcomes are illustrated in Table 4 below. Few home visits were conducted during the prescribed time period of the first 24 h after birth—only a quarter of the postnatal home visits occurred within the first 24 h, and the majority of visits that were intended to occur on the third or seventh day after birth (between 61 and 77%) occurred on the wrong day. The “wrong day” designation was used as an indicator of barriers to completing VHSG work as defined in the study protocol, where visits were expected to occur on the specific days as recommended by the WHO/UNICEF Joint Statement on Newborn Home Visiting.

Utilization of training materials appeared to be strong among both the VHSG and midwives at the health centers. Results indicate that a majority of VHSG volunteers utilized the training materials provided to them for their home visits (76.92%), and no individuals reported not using the materials. Additionally, health center staff reported a high level of training material utilization within the health centers (71.43%).

A large cadre of 504 VHSG workers were trained over the course of the stepped wedge trial, at monthly intervals over the course of 18 months. Likewise, a large number of midwives and cleaners, consisting of 113 HC staff were trained. Not all HC had cleaners on staff. All training activities were carried out as planned. Most HC staff trained were females (*n* = 98) compared with males (*n* = 15). By contrast, more VHSG volunteers trained were male (*n* = 274) than female (*n* = 230). Training sessions were conducted in the following 16 health centers: Prey Chour, Udamsorya, Leaybo, Angtasome, Kus, Tramkak, Prey Sleuk, Prambei Mum, Sronge, Rokakrao, Baray, Lim Chong, Rovieng, Tonle Bati, Vachieng, and Angkor Borey.

Several themes emerged from the qualitative data collection in relation to the implementation of the intervention components and training for those components (counseling during home visits). Interview topics from the data collection with VHSGs and mothers clustered around training experiences, conducting home visits and the logistical/social issues encountered during the intervention, and reception of the intervention by the target population. Major themes included: training quality, continuity of health center staff communication, and timing and ability to complete home visits.

### 3.1. Implementation of Training for VHSGs

Discourse of VHSGs related to the training volunteers received and how it strengthened their confidence to complete home visits, which they had never done before. One interviewee reflected, “[I] wasn’t sure that I could do the visits with the mother in the beginning, but I think the training was enough. (VHSG volunteer, 63 years old).”, “[I] learned about breastfeeding, newborn care, skin problems, danger problems, convulsions, normal movement of the baby, and cord care during the training. (VHSG volunteer, 53 years old).”

Within the training, volunteers spoke of what helped them comprehend and conceptualize the skills needed for visits. The flipchart was regarded as the most helpful tool when speaking with mothers and when learning new facts. Role-playing activities were perceived as a very useful component of the training. VHSG interviewees stated how role-playing was both the most effective and enjoyable component of their training days at the health center, “I liked the training a lot, as it taught me about the ten danger signs, which was very helpful, along with information on cord and skin care. Before I had trouble remembering the danger sings, especially the low birth weight threshold for the baby…but now I can remember it. The best part of the training was the role-playing, which helped me understand things better. (VHSG volunteer, 63 years old).”, “I learned about washing hands, breastfeeding, skin to skin contact, and a few danger signs…the training was enough. [I] like the role-playing the best. Also wrapping the baby was fun to learn. I had difficulty because I couldn’t note things down, as I don’t know how to write so [role-playing] helped with this. (VHSG volunteer, 28 years old).”

Several interviewees mentioned that having the trainings at the health center was inconvenient. Numerous volunteers were forced to travel via bicycle or foot to reach the trainings, which took time and effort. The health center was far from many of the participants, “The training was at the health center, so it was quite far for me. I took a bicycle to arrive there. (VHSG volunteer, 45 years old).”, “I usually go by foot or by bicycle...Sometimes [I] have to walk very far. (VHSG volunteer, 28 years old).”

The other critique was of the short duration and lack of a follow-up refresher training, after the intervention began. Volunteers spoke of the need for another training to assist them with any topic or technique that was harder to learn, “The training was only 2 days, so maybe it should be longer. (VHSG volunteer, 54 years old).”, “[I] feel very confident doing the home visits as [I] have been doing them for a while, but [I] wish there was a refresher training sometime from the NICCI team. I would also like more training on how to care for a newborn. (VHSG volunteer, 63 years old).”

### 3.2. Implementation of Home Visits and Counseling

Interviews conducted with the VHSG volunteers indicated that handwashing and bathing of the newborn were the most discussed topics with mothers. Participants reported that reducing the number of baths given to a newborn and avoiding bathing for the first 24 h to maintain a stable temperature for the newborn was challenging for families to accept. The following quotes illustrate the discussions over bathing with participants, “Before, mothers would bathe their babies many times, but now they only take a bath one or 2 times a day. (VHSG Volunteer, 50 years old).”, “The mothers think that right after the birth they should bathe the baby, so the mother doesn’t know that we need to avoid giving a bath to the baby right after birth. (VHSG volunteer, 62 years old).”

Similar to what was reported by the VHSGs, mothers who were interviewed also discussed reducing the frequency of bathing as a topic of home visit counseling, and also mentioned using warm water and immediate drying. Interviews indicated that women were given pertinent information on newborn care during visits, “[The VHSG] told me about the frequency of taking a bath for the baby and to use soap for the baby…She said that if taking a bath for the baby frequently, she is afraid the water will go into the lungs.(Mother, 31 years old).”, “[I] also gave a bath to the baby two to three times for the first baby, and only once per day now, as I learned this from the NICCI project.(Mother, 31 years old).”

Handwashing counseling was a consistent theme emphasized by every volunteer interviewed. Interviewees noted mothers and families often eschewed hand washing and relied on frequent bathing of newborns for hand hygiene. After visits, participants reported an increased understanding of recommended handwashing actions, as well as how to perform them. It was commonly reported in interviews that handwashing frequency, and knowledge regarding handwashing (how to wash hands, to use soap, when one should wash hands, etc.), increased, “The mother changes her behavior about washing her hands after this lesson. There was a culture of not washing hands when touching the baby. The mother asked “why do we need to wash our hands?” I explained that our hands are dirty and have bacteria after going to the bathroom. (VHSG volunteer, 62 years old).”, “The project has improved my knowledge and the mothers follow my advice about washing hands before touching the baby. The babies in the community never have problems [now]. (VHSG volunteer, 55 years old).”

Interviews with mothers also identified discussion of more frequent hand washing. One woman stated, “I now follow [the VHSG’s] advice about washing my hands before touching the baby. (Mother, 27 years old).”, “I tried to follow [VHSG] advice about washing hands before touching the baby. [We] need to wash hands three times, especially before touching the baby. (Mother, 23 years old).”

The interviews also indicated utilization of teaching tools and materials on handwashing by VHSG volunteers during home visits such as those discussed by participants. The most frequently utilized teaching aid was the flipchart. The flipchart was unanimously stated as the favorite of the teaching tools by both the volunteers and mothers. Flipcharts were frequently utilized to explain issues such as handwashing, bathing, and breastfeeding. The roleplaying technique was a well-received component of the VHSG training sessions.

#### 3.2.1. Perception of Home Visits

Volunteers reported satisfaction about their participation in the intervention, and felt the mothers had a strong appreciation of the work. Increasing mothers’ knowledge, learning about recognition of danger signs, and providing consistent support to the mothers were components of the program that interviewees felt were especially strong. The mothers also reported satisfaction with the intervention and counseling they received, “I think that I learned a lot from the visits. [The VHSG] told me about the frequency of taking a bath for the baby and to use soap for the baby and breastfeeding and she told me a lot more but I cannot remember. (Mother, 29 years old).”, “It’s not hard to do everything that the health center staff or the VHSG coordinator told me and I’m willing to do it because I really want my baby to grow up healthy. (Mother, 27 years old).”

One VHSG volunteer noted the change in practices over the intervening generation, “Thinking back at the time when I had young children, if there was a project like this, maybe one of my babies would not have passed away. I did not bring my child to the health center, I just treated the baby here and I didn’t know anything about this [counseling information]. (VHSG volunteer, 30 years old).”

Volunteers felt that this program helped changed the mothers’ behaviors, while increasing their health knowledge and awareness, “The mothers seem to understand how to care for the baby and interpret the danger signs more after this project. [The mothers] also seem to use the health center more, and [I think] this has lowered the illness, especially for preterm babies. (VHSG volunteer, 53 years old).”

Mothers’ attitudes towards the home visits and intervention were positive–none of the women interviewed indicated dissatisfaction with the program or the home visit component. Women reported that the VHSG workers helped them to increase their newborn health care knowledge, and that visits made them happy while also supporting better care of their children, “I felt comfortable when the VHSG came and learned a lot from the visit. It doesn’t bother me that the VHSG was a man. I felt comfortable and did not feel shy. I felt comfortable because of the benefit of having him come to give me the information. (Mother, 33 years old).”, “No, I didn’t know that the volunteer would come to visit [at that time], but the volunteer told me when she arrived at my house. Yes, it was a good surprise because she told us a lot and we didn’t know that much. (Mother, 27 years old).”

However, it is not clear whether mothers participating in the trial understood the distinction between the study data collection team and VHSGs. One mentioned a preference for the NICCI study coordinator who administered data collection forms, “[I] wish the coordinator came more often, as [I] really liked her. (Mother, 23 years old).”

Interview data from VHSG volunteers indicated that they perceived the intervention increased knowledge, while improving understanding in individual topics around newborn health. One volunteer stated, “I think the project has helped the mothers to know the danger signs of the baby. I am happy to help people, especially the babies. Even if I wasn’t a VHSG, I would still help people because now I have the knowledge so when I see a baby with danger signs, I can advise them to go to the health center because I have the knowledge now. The project has helped the mother know how to look after the baby and know about the danger signs. (VHSG volunteer, 45 years old).”

Mothers mentioned learning about danger signs, as well as general instructions regarding how to care for their child. However, many women reported they had not retained all the information provided by the visit. Most women could identify some danger signs, but numerous women stated they could not remember everything, “The VHSG came here two or three times and talked about the danger signs. I can only remember eye problem and fever... (Mother, 27 years old).”, “The [NICCI study] coordinator told me that a VHSG will visit me at home. This occurred at the time of delivery… He also gave me some information at the time of delivery. All I remember is how to keep the baby clean from the visit and some of what to look for [regarding danger signs]. (Mother, 30 years old).”

Interview participants also mentioned an increase in knowledge regarding care-seeking at appropriate facilities for newborn illness. The following statements illustrate this topic, “The VHSG coordinator advised me to bring the baby to the health center, and the baby had a very runny nose. They gave me some paracetamol at the health center, and the baby felt better. (Mother, 26 years old).”, “I think I learned a lot from the visits and I was advised that if I noticed any signs that were the same as in the leaflet or booklet or flipchart that I need to take the baby to the health center. (Mother, 29 years old).”

#### 3.2.2. Logistical and Social Issues Encountered During Implementation

VHSG volunteers reported various challenges to visiting families at home, which was the primary requirement for delivering the behavior change intervention components and for encouraging newborn care seeking following birth. Some volunteers described that it was difficult to locate family homes, and that they did not always feel comfortable approaching families at a sensitive time in their lives, when they did not know the family in advance. Hazards in the rainy season like impassable roads, along with other hazards such as menacing/stray dogs also presented barriers. VHSGs’ location relative to their community either facilitated or constrained access to mothers and families. Several women reported that they were visited very frequently since the volunteers lived within their community, “The volunteer did come to visit twice, but maybe many more because I sell fruit and vegetables and when she came to buy vegetables, she also did a check. The volunteer’s house is near here. (Mother, 27 years old).”, “[The VHSG] told me how to take care of the child and myself too. I can’t remember how many times she came, because she lives next to me so she comes over quite often. (Mother, 30 years old).”, “I notice marriages in the village, and a few months after the marriage I go to meet the woman and ask “are you pregnant? Or are you trying to be pregnant?” or sometimes I just go around the village and I see a woman who looks like she might be pregnant, so I ask. (VHSG volunteer, 45 years old).”, “The community is very big 500 families, 3000 people, and [the VHSG] mostly knows all the people except the young ones. Although the town is big, the people always pass by my home, or when he is grazing the cows, he is able to meet people. (VHSG volunteer, 62 years old).”

However, other mothers did not receive visits on time or at all. Some VHSG volunteers reported only making two visits, or missed visits if the health of the mother and newborn were good, “I travel by motorcycle or bicycle or foot to reach the mother’s houses. I will go in the afternoon, or a different day if I am busy. (VHSG volunteer, 62 years old).”, “Sometimes I only do 2 visits, as I see that the mother is healthy. I normally make her second visit on the third, and third visit on the seventh day, but sometimes I won’t make it as I am busy. (VHSG volunteer, 60 years old).”

Interviews illustrated that some VHSG volunteers did not adhere to the home visit schedule provided in the training but had to modify the timing of the home visits according to their own schedules, “Sometimes I forget to go for one of the visits to the mother. (VHSG volunteer, 53 years old).”, “Sometimes I am delayed with my own work, and can’t visit the mother. Sometimes, I go 2–3 months after birth. (VHSG volunteer, 52 years old).”

#### 3.2.3. Communication around Implementation of the Intervention

Health center midwives and VHSG volunteers described positive and frequent communication in connecting community members with health facilities to ensure participants received the care they needed, “When the health center staff call, I prepare my stuff and I go see the mother….. We have good communication with the health center, and they report to us if there is a problem. (VHSG volunteer, 28 years old).”

It was noted in interviews that midwives would seek out the VHSG volunteers to notify them about upcoming, or recent births, within their community. Communication between midwives and the VHSG volunteer was found to be mostly positive, “The midwife calls me to let her know that a woman is delivering, and there is no problem with this. I had already registered the mother though. (VHSG volunteer, 30 years old).”

Implementation was also aided by the communication facilitated through NICCI study staff coordinators, ensuring a continuum between community and facility, “I have good communication with the [NICCI staff] VHSG coordinator and before the training also there was already good communication, even though I wasn’t getting benefits yet. She was coming to check to see if there were pregnant women in the area and if there were pregnant women in my catchment area, she would let me know… the VHSG coordinator asks about my knowledge, and if there is something that I don’t know she explains step by step, like the handwashing or the danger signs. If there is a missed point, she will explain it to me. (VHSG volunteer, 60 years old).”, “The [NICCI] coordinator communicates with me every week to check up, although there is a formal checkup every 2 month. It is helpful to have a refresher on things I don’t know. (VHSG volunteer, 63 years old).”, “I have good communication with the VHSG coordinator. When I have problems, I call her. I am satisfied with our communication. We talk quite often. When we are in contact, we talk about the pregnant women and whether it is time for an interview or not, whether the women have given birth, etc. The VHSG coordinator helped me learn how to take the temperature, which I wasn’t sure how to do after the training. (VHSG volunteer, 50 years old).”

#### 3.2.4. Reception of the Intervention by the Target Population

Mothers felt comfortable with the VHSG volunteers due to their social status within the community. Their visits were perceived as beneficial. One interviewee stated, “I’m happy to welcome the VHSG coordinator even though she isn’t a relative. I’m happy to listen to her advice. (Mother, 31 years old).”

VHSG volunteers reported feeling that they were respected members of the community and took pride in their work, “I am a community member, so people know and respect [me].(VHSG volunteer, 53 years old).”, “[I] have no problems with any home visit because [I] am a well-known VHSG in the village, and am on the village council. (VHSG volunteer, 45 years old).”, “Everyone knows I am the elder women who works with pregnant women. I used to have a project where I would give the women a big gift when they became pregnant, so now people know to come and see me when they are pregnant. (VHSG volunteer, 53 years old).”

## 4. Discussion

The process evaluation collected qualitative and quantitative data to explore the implementation, context, and factors that may have constrained or contributed to the success of the NICCI intervention. Ultimately, the evaluation identified several factors that might have influenced the outcomes of the trial: continuity of health center staff communication, timing and ability to complete home visits, and training quality.

Training of health center staff and volunteers was implemented without issues, and quantitative data reflected this. Health center and volunteer communication appeared to function well, allowing for “handoffs” in this linked community–facility intervention. Mothers interviewed described learning about newborn care from the VHSG visits, though they could not always remember the full content of those visits. VHSGs reported that they felt their counseling had contributed to improved newborn care among the families that they worked with. However, the implementation was not without challenges.

Volunteers in the study reported a number of barriers including difficulty remembering training and the full content of the messages intended to be conveyed to mothers, along with logistical constraints to making home visits on schedule (including transportation and personal priorities). Volunteers who lived near their communities were more able to visit families as directed and on time (i.e., in the first 24 h after birth, as well as days three and seven of life) versus those who had to travel farther distances. The quantitative data provided information on the lower numbers of completed visits, where only a quarter of families received a visit on the first day of life. Quantitative data on this issue were complemented by explanatory qualitative data, exemplifying the mixed methods approach used for understanding implementation, wherein volunteers described transportation and other difficulties in completing visits on schedule.

Training provided in the intervention, an important component of implementation success, was considered appropriate with high satisfaction among participants, but could have been longer or included refresher elements, as described by the interviews with VHSGs. However, study data indicate that the materials provided during training were utilized as they had been intended to be, with 77% of VHSGs reporting utilizing these during home visits. Qualitative data echoed that the training sessions provided to VHSGs were beneficial, as participants described the utility of the sessions and finding them helpful.

This study has strengths and limitations. One strength is the depth and breadth of qualitative data gathered from in-depth interviews with participants who both provided and received implementation components. Another strength is the mixed methods approach, wherein quantitative monitoring data have provided details on implementation and resources available for the intervention, and qualitative data have provided information on the reasons for these outcomes.

Nonetheless, a limitation of the study is the relatively limited amount of quantitative data available to contribute to understanding the barriers and facilitators to the implementation of the trial. Another potential limitation is social desirability or reporter bias, likely to be particularly strong in the study setting, which may have hindered attempts to gain an accurate picture of the implementation process. There was noticeable lack of any negative comment about the intervention by participants in this study. In several instances, interviewees were asked what critiques they have regarding the following components of the program: training, house visits, communication, and supervision. Throughout the VHSG interviews, there was a trend of not answering questions in a manner that may have seemed negative. Interviewees either had no response or would speak positively regarding the topic. Most answers focused on the ability to fix a problem or complete the task. Mothers interviewed were also reluctant to provide critical feedback for reflection. This may have hampered the evaluation of challenges or obstacles to the implementation, and is likely related to cultural norms.

Few process evaluations of newborn intervention studies have been conducted to understand the implementation of these types of trials. One process evaluation of a trial for newborn survival in Vietnam identified several salient factors related to the implementation of the trial: continuity of intervention groups’ work, adequate attributes and skills of facilitators, and targeting problems along a continuum of care [13]. Another process evaluation from Nepal highlighted the need to reduce complexity and the number of messages being delivered by volunteers to mothers and families around newborn health [22]. Given that mothers reported difficulty in remembering the full extent of information provided by VHSGs in this study, it is likely that the current study might have benefitted from reducing the number of messages delivered by volunteers as well.

Future research must explore the sustainability of utilizing community volunteers [23], and it would be important to gauge how volunteer engagement may be greater in the presence of supportive supervision and more extensive training, as well as exploring motivation [24]. The current study results indicated that VHSGs were unable to carry out home visits on the scheduled days of newborn life, due in part to factors that could not be avoided such as transportation and time difficulties. Their own primary (paid) employment in other occupations must be considered, given that volunteers, even those receiving incentives, would inevitably need to prioritize their own personal health, jobs and families over volunteer requirements in some circumstances.

Additionally, the role of study staff is an important factor to explore as intervention trials are considered at scale. In the current study, participant families may not have always understood the difference between study staff and VHSG volunteers’ roles, and in some cases preferred to engage with study staff. Ultimately, it is unclear whether utilizing volunteers with low levels of reimbursement, and without extensive supervision or other resources, will result in sustainably improved outcomes, and indeed this has been debated recently in the context of newborn care interventions [25,26]. The current process evaluation highlighted constraints to implementation, and also the need to attend to staffing, training, and ensuring the quality of newborn health interventions in planning for the scaling-up of newborn survival programming, which should be considered in future activities.

## 5. Conclusions

The current implementation study utilized process evaluation to investigate and explore the barriers and facilitators of the intervention trial. Results highlighted constraints to the implementation of a large community-based trial in this lower middle-income setting, and also the need to attend carefully during studies to staffing, training, and ensuring the quality of newborn health interventions. The results of this study suggest recommendations for future newborn health research in the country and in the region, notably that implementation by staff other than volunteers be explored where feasible. Additionally, it is recommended that the appropriate timing of home visits, particularly in the first 24 h of birth, be highlighted as a crucial component of any future newborn health study. Finally, a continued focus on quality of care especially as related to infection prevention control and water, sanitation, and hygiene, in health facility settings will be key in future projects aimed at ensuring the health of newborns. The results may aid in planning for the scaling-up of newborn survival programming, particularly in the Southeast Asian setting, which should be considered in future research.

## Figures and Tables

**Table 1:** Characteristics of interview participants: village health support group (VHSG) volunteers (*N* = 13).

**Occupation**		***n***
	Farmer	3
	Vendor	4
	Farmer and government salary	2
	Multiple occupations	4
**Village**		
	Roveang	3
	Prambey Mum	2
	Ankor Borey	2
	Tonle Bati	1
	Ang Ta Som	1
	Srok Chey	1
	Ang Kmao Tong	1
	Hoi Leap	1
	Roka Krow	1
**Gender**		
	Male	3
	Female	10
**Marital Status**		
	Married	9
	Divorced	1
	Widowed	2
	Single	1
**Religion**		
	Buddhist	13
**Ethnicity**		
	Khmer	10
	Cambodian/Other	3
**Other**		**Mean (Range)**
	Age, in years	47 (28–63)
	Years of education	5 (0–7)
	Years working as a VHSG	8 (1–23)

**Table 2:** Characteristics of focus group discussion (FGD) participants: midwives (N = 15).

**Training**		***n***
	Primary (1 yr training program)	6
	Secondary (4 yr training program)	9
**Health Center**		
	Tonle Bati	1
	Ankor Borey	1
	Prambay Mum	2
	Preyslek	2
	Rokraro	5
	Laybor	2
	Roveang	2
**Gender**		
	Female	15
**Marital Status**		
	Married	9
	Divorced	1
	Widowed	1
	Single	2
	N/A	2
**Religion**		
	Buddhist	14
	N/A	1
**Ethnicity**		
	Khmer	14
	N/A	1
**Other**		**Mean (Range)**
	Age, in years	32 (25–47)
	Years of education	11 (8–12)
	Years working as a midwife	9 (3–37)
	Years working in that health center	8 (1–37)

**Table 3:** Characteristics of interview participants: mothers (*N* = 15).

**Age, in Years**	***n***
<25	5
25–30	8
>31	2
**Education, in Years**	
<5	2
5–10	10
>10	2
**Occupation**	
None	3
Garment worker	4
Multiple occupations	5
Other	2
N/A	1
**Health Center**	
Leay Bor	3
Roveang	3
Prambay Mum	2
Roka Kraw	1
Tonle Bati	1
Saman	1
Ankor Borey	1
Prambay Mum	1
Phung Tram	1
N/A	1
**Gender**	
Female	15
**ID Poor Card**	
Yes	3
No	12
**Relatives/Family Living Nearby**	
Yes	3
No	12
**Family Asked to Clean the Delivery Room after Most Recent Birth**	
Yes	10
No	5
**Family Asked to Clean the Delivery Room after Previous Birth**	
Yes	7
No	3
N/A	5
**Contraceptive Use**	
Yes	2
No	11
Plans to use	2

**Table 4:** Process evaluation indicators and outcomes.

**Primary Outcomes**	***n*/Percentage**
Number of VHSG volunteers trained	504
Number of health center staff trained (midwives and cleaning staff, where cleaners were on staff)	113
Newborns visited by a VHSG volunteer within 24 h of birth	25.67%
**Newborns Visited by a VHSG Volunteer on Day Three of Life**	
Yes	15.38%
Yes, but on wrong day	76.93%
No answer	7.69%
**Newborns Visited by a VHSG Volunteer on Day Seven of Life**	
Yes	15.38%
Yes, but on wrong day	61.54%
No answer	23.08%
**Utilization of Training Materials as Part of VHSG Equipment**	
Yes	76.92%
No	0
No answer	23.08%
**Utilization of Training Materials in Health Centers**	
Yes	71.43%
No	0
No answer	28.57%
